# Nutrition Module: Addressing the Nutrition Education Gap in Undergraduate Medical Curricula via a Novel Approach

**DOI:** 10.1007/s40670-024-02114-9

**Published:** 2024-07-15

**Authors:** Pinyu Chen, Seth McKenzie Alexander, Vanessa Baute Penry

**Affiliations:** 1 Wake Forest University School of Medicine, Winston-Salem, NC USA; 2 Department of Health Sciences, The University of North Carolina School of Medicine, Chapel Hill, NC USA; 3 Department of Neurology, Atrium Health Wake Forest Baptist Medical Center, Winston-Salem, NC USA; 4 Atrium Health Wake Forest Baptist Medical Center, 1 Medical Center Blvd, Winston-Salem, NC 27157 USA

**Keywords:** Nutrition, Online module, Medical education

## Abstract

**Introduction:**

Despite the known importance of nutrition on health outcomes, most medical curricula do not dedicate sufficient time to nutrition topics. Many barriers prevent the successful integration of nutrition education into existing curricula.

**Methods:**

We created an online nutrition module to educate students about foundational nutritional topics. To assess the efficacy of the module and improve integration of knowledge, students were asked to take a pre-assessment and a post-assessment immediately before and after completion of the module. Two months after completion, students were asked to take a follow-up assessment to assess long-term retention of the information covered in the module.

**Results:**

A total of 15 medical students completed all the requirements of the nutrition module (including pre-, post-, and follow-up assessments). The mean percent correct on the pre-, post-, and follow-up assessments were 67.5%, 87.0%, and 83.5%, respectively. The absolute difference between the pre- and post-module scores was 3.8 points (19.0%, *t* = 9.2, *p* < 0.0001). The absolute difference between the mean post- and follow-up scores was − 0.93 points (4.7%, *t* =  − 1.7, *p* = 0.1154).

**Discussion:**

Most medical students do not feel adequately prepared to counsel patients on nutrition. Development of an accessible, online nutrition module was effective in teaching medical students about nutritional topics and in retaining the information over time. Advantages of the module include flexibility for students to choose when to complete the learning, brief (< 1 h) concise material, and the ability for educators to quickly update the module content.

**Supplementary Information:**

The online version contains supplementary material available at 10.1007/s40670-024-02114-9.

## Introduction

Despite the known importance of nutrition in the prevention and management of chronic diseases, comprehensive nutrition education is lacking in medical schools, and many graduating physicians are not prepared to address the nutritional concerns of their patients [1–3]. The National Research Council (NRC) recommends at least 25 h of dedicated nutrition curricula; however, a mere 30% of medical students will graduate having met this recommendation [4]. On average, 19 h of medical curricula are dedicated to nutrition education, and this has decreased over the last decade [5]. In 2023, the Accreditation Council for Graduate Medical Education hosted a summit to discuss the current state of medicine as it relates to nutrition, with the conclusion that diet-related diseases are the most prevalent causes of illness in the United States. Suggestions were made to integrate nutrition into existing medical school coursework to teach students about the relationship of nutrition to health and how it acts as a social determinant of health [6].

In fact, 79% of medical school instructors agree that students need more nutrition instruction, and that current nutrition education is not sufficient to build student confidence in delivering nutrition counseling [1, 7]. Only 18% of medical schools nationwide require a nutrition course, and among those, there is no set standard in the content taught and how it is delivered [3, 5]. Even in review books for the US Medical Licensing Examination (USMLE), the content is focused on vitamin and mineral deficiencies in relation to conditions, such as scurvy, and not clinical nutrition related to prevention of chronic diseases [8, 9]. For example, discussions on how to manage cardiovascular conditions (i.e., type II diabetes, hyperlipidemia) with dietary modifications is lacking. In a 2017 study, 85% of medical students in their clinical years indicated they were not confident in their preparedness to counsel patients on nutrition topics, and 86% indicated they would be interested in further practical, evidence-based nutrition education [10].

Physicians cite many barriers that prevent them from delivering nutrition counseling services [11–15]. In 1995, Kushner et al. surveyed primary care physicians nationwide about their perceived barriers to nutrition counseling. Of the physicians that responded, the 4th and 5th most commonly encountered barriers were lack of training in counseling skills and a deficit of knowledge about nutrition [12]. The top barrier was lack of time with patients [12]. In addition, physicians do not feel confident in their perceived ability to positively influence the lifestyle and eating habits of patients [14]. A follow-up study in 2010 demonstrated nutrition counseling continues to be lacking among today’s physicians [11, 12]. Fortunately, there is increasing recognition among physicians of the importance of possessing the knowledge and ability to counsel patients about nutritional lifestyle changes [6, 16–18]. Studies have also shown that physicians who personally practice healthy habits are more likely to spend more meaningful time counseling patients [18, 19]. Of note, links have been found between dietary habits and mental health status [20]. With high burnout rates and stress facing medical students and health providers, it is worth exploring if acquiring nutrition knowledge correlates with personal mental health benefits in students [21].

Multiple barriers hinder the ability to formally incorporate nutrition into medical school curricula, such as limited curricular time, lack of funding, and qualified instructors [10, 22]. In the mentioned 2017 study, the proposed nutrition elective was not sustainable due to a lack of facilitators to maintain the program [10]. Our theoretical framework relies on the fact that online modular learning has long been used in medical training as a method for teaching new concepts to medical students, with proven efficacy [23–25]. In a leukemia learning module, the authors used pre- and post-test assessments immediately preceding the module and 2 weeks after completion to determine its efficacy. The module was designed with interactive questions and activities [23]. Another online module aimed to teach students about wound care through the use of knowledge checks, pictorial examples, and videos, with proven success as well [25].

Hence, online modular learning can be an efficacious, cost-efficient, and sustainable way of teaching medical students about nutrition that gives students the flexibility to complete on their time. For example, the Department of Nutrition at the University of North Carolina at Chapel Hill created an online module called the *Nutrition in Medicine Project*. The project delivers nutrition curriculum to their medical students without needing faculty to invest ongoing teaching time [2, 26]. In another example, Rutgers Robert Wood Johnson Medical School utilized a 30-min online nutrition education module to help medical students identify signs of malnutrition in hospitalized patients. After completion of the module, students demonstrated an improvement in their knowledge base [27]. Other existing nutrition modules are not in an online format and require multiple hours of instruction in-person over several days [28]. While in-person lecture formats may be effective teaching tools, their delivery format may not be a sustainable solution to existing curriculum barriers given the need for dedicated classroom time. The existence of other publications working to tackle the lack of nutrition education in medical curriculum strongly supports the necessity for change to occur. However, the majority of the publications focus on in-person didatic sessions that also require dedicated faculty teaching the material.

The online nutrition education module, entitled *Foundations in Nutrition*, is distinct from other existing modules as it is based on specific student knowledge gaps including foundational nutrition topics, taught through interactive exercises, case-based learning, and nutrition counseling approaches. The purpose of this study is to determine the efficacy and impact of teaching medical students about fundamental nutrition topics in about 45 minutes via a flexible, online format. It utilizes active learning to improve student learning and retention [29]. The pre- and post-assessment structure is also intentionally designed given the module’s structure to encourage retention through the testing effect [30]. The online module can be completed in less than an hour, at any time, to accommodate the existing, demanding schedule of medical students, and the interface allows for quick and easy updates to the content as nutrition research evolves.

## Methods

### Development

The development of *Foundations in Nutrition* was based on content identified as lacking according to a needs assessment survey (ESM Appendix 1) sent out via email to currently enrolled medical students. The questions were guided by suggestions for writing an effective survey [31]. On the survey, students were asked to indicate their confidence and preparedness in counseling patients about their nutritional intake, as well as a series of questions pertaining to their knowledge base of foundational nutrition topics. Questions were based on commonly encountered clinical scenarios and anecdotal statements from medical students about their nutrition knowledge. The survey was voluntary, and responses were anonymized.

*Foundations in Nutrition* was created using Rise 360 Articulate (Articulate Global LLC, New York, NY) by medical students and physician-educators trained in nutrition. The module highlights the pertinent clinical nutrition information based on the local standard of care and common chronic illness dietary counseling, *Dietary Guidelines for Americans, 2020–2025*, and a PubMed literature search of the nutritional benefits of vegetables, fibers, fats, fruits, grains, dairy, and protein [32]. The nutrition module features embedded interactive exercises, short example video, case study examples, and brief knowledge checks within the lessons to promote active learning. Each individual lesson contains checkpoints (i.e., quizzes, sorting exercises, and review flashcards) within the material to encourage interactive and active recall of the content, synthesize what they just learned, and identify the gaps in their knowledge. To enhance retention, the last lesson in the module is a case study designed to help the learners walk through a real-life clinical application of all the material presented in the previous lessons. In total, the module is divided into 12 lessons and was designed to take no longer than an hour to complete. The learning objectives for the module are:

- Understand the nutritional importance of consuming carbohydrates, vegetables, fibers, fats, fruits, grains, and protein.
- Know common examples of carbohydrates, vegetables, fibers, fats, fruits, grains, and protein.
- Know the recommended daily intake of carbohydrates, vegetables, fibers, fats, fruits, grains, and protein.
- Understand the common barriers that individuals face to eating a healthy diet and know the recommendations to overcome them.
- Be able to read a nutrition label and understand what is included in the food product.
- Be able to advise patients on healthy alternatives to their everyday foods.

### Implementation

Participation was voluntary and open to all currently enrolled medical students at a large, private medical school, regardless of whether they had previous formal training or experience in clinical nutrition counseling. They were emailed the module and given the freedom to complete the module wherever and whenever they chose, with the only restriction being that they complete the pre- and post-module assessments on the same day.

### Assessments

The pre- and post-module assessments were embedded directly within the module at the start and end of the module, respectively. Two months after completing the post-module assessment, participants were emailed the follow-up assessment and asked to complete it within the next 7 days. Students were not shown the correct answers to the 20 content questions after completing any of the assessments. The questions were validated by physicians who have training in medical education and nutrition counseling. Descriptive and comparative statistical analyses were completed using STATA BE version 17.0 (StataCorp LLC, College Station, TX). All comparative statistical testing was completed using paired *t*-tests with a level of statistical significance (*⍺*) set at 0.05.

#### Pre-Module Assessment

Prior to starting the module, the students completed a pre-module assessment (ESM Appendix 2) composed of 20 questions based on content within the nutrition module that was most relevant and frequently encountered in the clinical setting. These questions were different from the checkpoint questions that are embedded within the module. In addition to the content questions, students were asked to indicate on a sliding scale how prepared they currently felt about talking to patients in clinical settings about nutrition (with 0 being not prepared to 100 being very prepared). They were also asked to indicate their confidence in talking to patients in clinical settings about nutrition (with 0 being not confident to 100 being very confident).

Seven questions from the Mayo Clinic Well-Being Index (MSWBI) were included on the pre- and follow-up module assessments to determine if learning about nutrition topics would influence students’ mental health and resiliency [21, 33]. This survey was selected because it has been validated by research and was designed specifically for use in medical students to assess for fatigue, burnout, and quality of life [34]. The responses were scored according to the guidelines set by the MSWBI out of 7 points.

#### Post-Module Assessment

Immediately after completion of the module, students were directed to take the post-module assessment (ESM Appendix 3) containing the same 20 content questions found in the pre-module assessment. The post-module assessment, required to be taken on the same day as the pre-module assessment, did not include the MSWBI.

#### Follow-up Module Assessment

The follow-up assessment (ESM Appendix 4) was distributed 2 months after the completion of the module, and students had a week to complete it. This timeframe was chosen to determine whether students retained the information learned in the module. The follow-up assessment contained the same questions as the pre-module assessment, including the MSWBI. In addition, there were questions asking students to indicate, on a Likert scale, information about potential changes in their dietary habits as a result of the module. These questions were based on literature identifying a relationship between dietary habits and mental health, as well as how a physician’s own healthy habits can influence patient’s willingness to change [33, 35].

## Results

A total of 65 (participation rate, 11.2%) medical students (20 from the fourth-year class, 19 from the third-year class, 13 from the second-year class, 13 from the first-year class) completed the preliminary needs assessment survey. 27.7% (18 of 65) of the responders had prior experience with nutrition counseling. As shown in Table 1, 12% of students strongly agreed that they felt confident in their ability to counsel patients, and 14% of students strongly agreed that they felt confident in their ability to answer patient questions.

**Table 1:** Student responses from needs assessment survey

	Strongly agree	Agree	Neutral	Disagree	Strongly disagree
I feel confident in my ability to answer patient questions on nutrition recommendations and dietary intake. (*n* = 65)	9 (14%)	32 (49%)	10 (15%)	9 (14%)	5 (8%)
I feel confident in my ability to counsel patients on nutrition recommendations and dietary intake. (*n* = 65)	8 (12%)	27 (42%)	16 (25%)	10 (15%)	4 (6%)

**Fig. 1:**
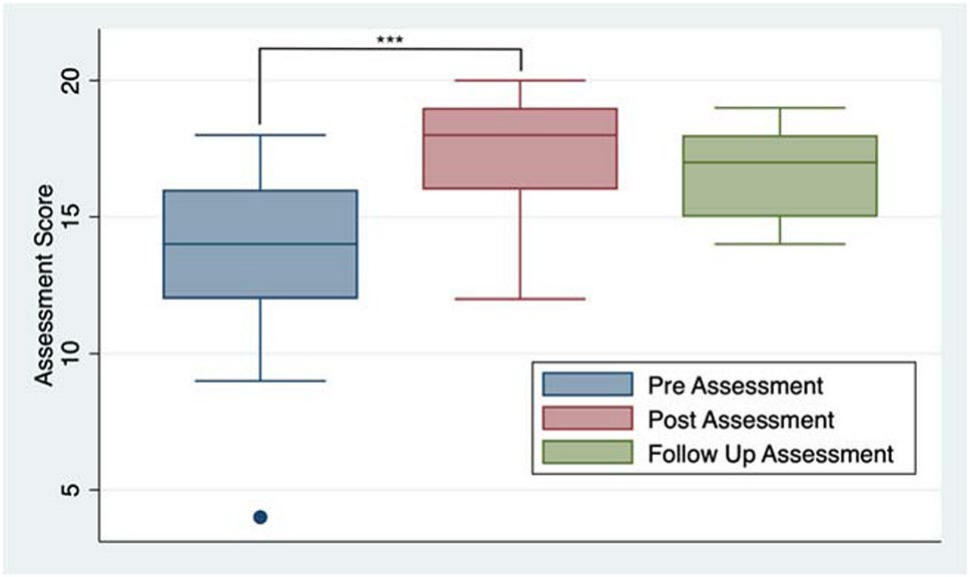
Box plot of the assessment scores throughout the intervention (*** indicates a *p*-value < 0.0001)

Twenty-nine (participation rate, 5%) individuals completed the pre-module assessment, with the mean correct score being 13.5 (67.5%, SD 3.0) out of 20 questions (Table 2). None of these 29 individuals had any previous formal training or experience in clinical nutrition counseling. Out of those 29 individuals, only a total of 20 individuals completed the post-module assessment, with a mean correct score of 17.4 (87.0%, SD 2.3). These 20 medical students (three from the first-year class, 11 from the second-year class, five from the third-year class, and one from the fourth-year class) completed the pre- and post-module assessments, including the nutrition module. The difference between the mean pre- and post-module scores was 3.8 points (19.0%, *p* < 0.0001), representing a statistically significant increase in the scores (Fig. 1). Five of these participants (25%) were lost to follow-up 2 months later; therefore, a total of 15 individuals completed the follow-up assessment, with a mean correct score of 16.7 (83.5%, SD 1.9). The difference between the mean post- and follow-up scores was − 0.93 (4.7%, *p* = 0.1154), which was not a statistically significant decrease.

**Table 2:** Performance on assessments

Assessment scores	Mean	% correct
Pre (*n* = 29)	13.5	67.5
Post (*n* = 20)	17.4	87.0
Follow-up (*n* = 15)	16.7	83.5

From the pre-module assessment, students (*n* = 29) indicated their mean preparedness and confidence level to counsel patients on nutrition to be 41.4 and 36.9, respectively. From the follow-up module assessment, students (*n* = 15) indicated their mean preparedness and confidence levels to be 69.2 and 67.0, respectively. Preparedness level significantly increased by 25.93 (*p* = 0.0001), and confidence level significantly increased by 28.13 (*p* < 0.0001). Of the students that completed the follow-up assessment, 53.3% (8 of 15) agreed that they changed their dietary habits because of the nutrition module. The remaining students were either neutral (33.3%, 5 of 15) with the statement or disagreed (13.3%, 2 of 15). Overall, 93.3% (14 of 15) of the students agreed or strongly agreed that the nutrition module positively influenced their dietary habits. For the MSWBI, on average, students’ scores were 2.3 in the pre-module assessment and 2.7 in the post-module assessment (*t* =  − 1.2, *p* = 0.24).

## Discussion

Despite the growing awareness of the importance of nutrition education for medical students, there remains a paucity of nutrition content in the medical curricula [5]. With the increasing prevalence of chronic diseases that can be prevented or managed with nutrition interventions, such as heart disease and diabetes, it is imperative that medical students have the knowledge to counsel patients on their dietary habits [36–38]. Based on the results of our needs assessment survey and review of the literature, the majority of medical students do not feel confident with their current nutrition counseling skills [1, 10, 39].

The goal of creating *Foundations in Nutrition* was to develop an impactful, low-cost, sustainable, and accessible form of nutrition education for medical students that can be used at other medical schools. Furthermore, the module was purposely written in layman’s terms to make it easily comprehensible for all learners and with patient-centered language in mind so the knowledge learned is easily transferable to counseling skills. The significant improvement in assessment scores between the pre- and post-module assessments highlights that students gained nutritional knowledge with the module. There was no statistically significant decrease in the follow-up score 2 months after completion of the module, indicating that students retained the information over time. As seen in Fig. 1, there was less variability in scores seen during the follow-up assessment compared to the post-module assessment. This may reflect learner retention and the fact that the students had time to synthesize the information and develop an understanding of the material rather than relying on recency bias to answer the questions. Our results verified that the educational content delivery method (i.e., interactive exercises, short example video, case study examples, and brief knowledge checks) not only promoted active learning but also helped students retain the information long-term.

Our intervention addressed the current barriers to successful longitudinal integration of nutrition education into medical curricula. Specifically, the lack of time to schedule more content into the curriculum and lack of funding to hire dedicated faculty to teach and maintain a nutrition curriculum. While nutrition is listed as one of the competencies in USMLE Content Outline, the topics are explored mainly in a micronutrient context with vitamin and mineral deficiencies and rarely in discussion on a macronutrient context with carbohydrates or proteins [8, 9, 40, 41]. Most importantly, the nutrition topics are not discussed in a clinical context that prepares medical students to counsel patients on their dietary habits. Despite the module not being a required component of our medical curricula, students still effectively learned from the module and retained the information over time. Furthermore, the flexibility to complete the module in their own free time did not interfere with their ability to learn from the module and was effective in increasing their knowledge base. The online module can also be easily updated through the hosted platform as medical education demands, nutrition research, and student feedback change over time, with changes reflected immediately. There is no need for dedicated faculty to secure funding for protected time to plan coursework and give lectures. From a review of online literature, existing solutions to the lack of medical education in medical curricula mainly involves in-person didactics that require dedicated faculty to teach. However, this requires carving time out of the already strenuous schedules of medical students and faculty to find a time for everyone to meet. *Foundations in Nutrition* is an efficacious, cost-efficient, and sustainable way of teaching medical students about nutrition, making it a potential solution to overcome these curricular barriers.

While students indicated that their dietary habits were positively influenced after completing the module, there seems to be no impact on their well-being. The MSWBI scores indicated that there was no significant improvement in the mental well-being of medical students from completing the module. However, there might be confounding variables that affected student responses, such as too few students to gauge an impact and monthly variations in course load, rotations responsibilities, and perceptions of stress. Furthermore, nutrition is one of several factors that may influence an individual’s well-being, so, in isolation, it may not shift the well-being score.

A limitation of our nutrition module includes difficulty recruiting medical students to complete an additional elective “assignment” with their busy schedules, resulting in a smaller sample size albeit statistically significant. Students that participated in the study might have completed the module because of existing interests in nutrition that inclined them to learn more, which could bias the post- and follow-up scores to be higher. The low recruitment numbers further reinforce the need to incorporate nutrition into the curriculum formally because most medical students have a paucity of nutrition knowledge. Strengths of *Foundations in Nutrition* include ease and flexibility of use for medical students, the ability to quickly maintain and update the module over time as deemed necessary, and the brevity of the curriculum. To the best of our knowledge, *Foundations in Nutrition* is the only existing nutrition module presented in an online format designed to equip busy medical students with the tools to counsel patients about nutrition.

Because of the low sample size, it would be important to repeat this study in the future with a larger sample size perhaps across several medical schools. Potential ways to encourage students to complete the study would be to offer incentives for completion or gamification of learning to see which class year has the highest completion rates. Further directions for exploration include making another version of the module with shortened lessons to make it easier for students to digest while having comparable efficacy. The information could also be converted to a small handbook that students can reference easily with patients. Another potential route is to deliver the module content, including assessments, in a traditional lecture format in order to compare the effectiveness of using an online module to teach medical students versus an in-person lecture. This could be as simple as incorporating a short session in clinical skills courses for talking to patients about their nutrition habits. Occasionally, experiential learning sessions, such as teaching kitchens, could be offered as an adjunct to students. Most importantly, as iterations of the module are created, feedback from medical students will be collected to improve the module to better fit their needs and interests.

A version of *Foundations in Nutrition*, containing the same content and checkpoint activities, has been made without the pre- and post-assessments embedded directly into it. The authors hope that other medical schools will utilize our module in their curriculum to introduce clinical nutrition and its importance to medical students. The module is meant to serve as starting ground for other schools to begin their own process of incorporating nutrition into their curriculum as it fits the needs of their own students. While we believe our novel, online module is an effective solution to overcome existing curriculum barriers, there is still a need to explore other avenues of education because this is a complex issue with a variety of solutions that could be tailored to fit the needs of each medical institution.

## Supplementary Information

Below is the link to the electronic supplementary material.

Supplementary file1 Needs Assessment Survey (PDF 62 KB)

Supplementary file2 Pre-Assessment (PDF 188 KB)

Supplementary file3 Post-Assessment (PDF 86 KB)

Supplementary file4 Follow Up Assessment (PDF 180 KB)

Supplementary file5 Nutrition Module (DOCX 15 KB)

## Data Availability

The data in this study is available upon request made to the corresponding author, provided this request is made in compliance with federal privacy guidelines regarding educational data.
