# Supplementary material for: Nutrition Module: Addressing the Nutrition Education Gap in Undergraduate Medical Curricula via a Novel Approach
Source: Med Sci Educ. 2024 Jul 15;34(6):1361–7. doi: 10.1007/s40670-024-02114-9 (PMC11699193; doi:10.1007/s40670-024-02114-9)
Supplement: Supplementary file 1 — Needs Assessment Survey (PDF 62 KB) [file 40670_2024_2114_MOESM1_ESM.pdf]

Needs Assessment Survey

We are taking steps to address the current gaps in clinical counseling (i.e., nutrition) education, and your answers will help us improve our curriculum.

Your answers will remain completely anonymous, and will not affect your grades, performance evaluations, or assignments.

Thank you!

---

1) What is your class year?

- ◯ Class of 2022
- ◯ Class of 2023
- ◯ Class of 2024
- ◯ Class of 2025

---

2) Prior to medical school (degree, career, etc), did you have training or background in lifestyle intervention topics? Select all that apply.

- ☐ Nutrition
- ☐ Exercise
- ☐ Mindfulness
- ☐ None

---

3) Thinking on your clinical experiences in the past 12 months, which of the following lifestyle intervention topics have you advised patients on? Select all that apply.

- ☐ Nutrition
- ☐ Exercise
- ☐ Mindfulness
- ☐ None

4) On average, how often do you counsel your patients about their nutrition habits in a given week?

- ◯ 0% of patients
- ◯ 25% of patients
- ◯ 50% of patients
- ◯ 75% of patients
- ◯ 100% of patients

5) How often do you think a patient could benefit from nutrition counseling but end up not receiving it?

- ◯ 0% of patients
- ◯ 25% of patients
- ◯ 50% of patients
- ◯ 75% of patients
- ◯ 100% of patients

Please answer the following questions in regards to nutrition.

6) How prepared you felt about talking to patients in clinic about these topics for the first time?

Not Prepared — Very Prepared

(Place a mark on the scale above)

7) How well do you think the current Wake Forest School of Medicine curriculum prepares you for advising patients on these topics?

Not Well — Very Well

(Place a mark on the scale above)

8) How comfortable do you feel about advising patients on these topics?

Not Comfortable — Very Comfortable

(Place a mark on the scale above)

9) How confident are you that your dietary habits meet the recommended daily nutritional intake?

Not Confident — Very Confident

(Place a mark on the scale above)

Please rate how strongly you agree or disagree with each statement.

10) I feel confident in my ability to counsel patients on nutrition recommendations and dietary intake.

- ◯ strongly disagree
- ◯ disagree
- ◯ neutral
- ◯ agree
- ◯ strongly agree

11) I feel confident in my ability to answer patient questions on nutrition recommendations and dietary intake.

- ◯ strongly disagree
- ◯ disagree
- ◯ neutral
- ◯ agree
- ◯ strongly agree

12) I know the recommended daily servings of fruit and vegetable intake.

- ◯ strongly disagree
- ◯ disagree
- ◯ neutral
- ◯ agree
- ◯

13) Nutritional intake impacts chronic medical conditions.

- ◯ strongly disagree
- ◯ disagree
- ◯ neutral
- ◯ agree
- ◯ strongly agree

14) I am aware that patients face internal barriers (i.e., lack of motivation or knowledge) that prevent them from receiving adequate nutritional intake.

- ◯ strongly disagree
- ◯ disagree
- ◯ neutral
- ◯ agree
- ◯ strongly agree

---

15) I am aware that patients face external barriers (i.e., lack of accessibility or affordability) that prevent them from receiving adequate nutritional intake.

- ◯ strongly disagree
- ◯ disagree
- ◯ neutral
- ◯ agree
- ◯ strongly agree

---

16) I think it is important for physicians to be able to counsel patients on nutrition interventions.

- ◯ strongly disagree
- ◯ disagree
- ◯ neutral
- ◯ agree
- ◯ strongly agree

---

17) My own health habits may impact the health habits of my patients.

- ◯ strongly disagree
- ◯ disagree
- ◯ neutral
- ◯ agree
- ◯ strongly agree
