# Supplementary material for: Nutrition Module: Addressing the Nutrition Education Gap in Undergraduate Medical Curricula via a Novel Approach
Source: Med Sci Educ. 2024 Jul 15;34(6):1361–7. doi: 10.1007/s40670-024-02114-9 (PMC11699193; doi:10.1007/s40670-024-02114-9)
Supplement: Supplementary file 2 — Pre-Assessment (PDF 188 KB) [file 40670_2024_2114_MOESM2_ESM.pdf]

Pre-Module Assessment

Thank you for your participation in this research study!

Taking part is voluntary. You may refuse to participate or withdraw from the study at any time and for any reason without affecting your grades, performance evaluations, or assignments. You will not be pressured into participating in this research study by any statements or implied statements that your grades, performance evaluations or assignments will be affected by your willingness to enroll in the study.

Create a unique study ID based on the model of your car and the last 4 digits of your cell phone number in all lowercase (e.g., camry8378). This will not be used to link your identity.

Please keep track of your study ID as we will ask you for it each time you complete an assessment.

1) Study ID

____

2) What is your class year?

- ◯ Class of 2022
- ◯ Class of 2023
- ◯ Class of 2024
- ◯ Class of 2025
- ◯ Class of 2026

3) Do you have any prior, formal training or experience in clinical nutrition counseling (i.e., registered dietitian, nutritionist)?

- ◯ Yes
- ◯ No

Select the best answer choice for assessment questions 4-23 without using resources.

4) What does the glycemic index of a carbohydrate tell you?

- ◯ How sweet the carbohydrate is
- ◯ The ratio of fiber to sugar in the carbohydrate
- ◯ How quickly the carbohydrate will cause an increase in blood glucose levels
- ◯ The ratio of fructose to glucose

5) For which of the following set of patients is it especially important for them to understand glycemic index?

- ◯ Patients with sleep disorders
- ◯ Patients older than 60 years
- ◯ Patients with diabetes
- ◯ Patients with high blood pressure

6) For a product to be marked 100% whole grain, what is the minimum requirement for grams of grain per serving?

- ◯ 11g
- ◯ 16g
- ◯ 21g
- ◯ 26g

7) Which of the following is NOT an example of polyunsaturated fats?

- ◯ Salmon
- ◯ Avocado
- ◯ Almonds
- ◯ Flaxseed

8) White bread is an example of a carbohydrate with high glycemic index.

- ◯ True
- ◯ False

9) Frozen fruits contain less nutritional value than fresh fruits.

- ◯ True
- ◯ False

10) It is recommended to choose foods that are:

- ◯ Higher in %Daily Value for Dietary Fiber and Vitamin D
- ◯ Higher in %Daily Value for Calcium, Iron, and Potassium
- ◯ Lower in %Daily Value for Saturated Fat, Sodium, and Added Sugars
- ◯ All of above

11) The benefits of consuming fiber include:

- ◯ Preventing diarrhea
- ◯ Weight gain
- ◯ Lowering the risk of heart disease
- ◯ Increasing your HDL cholesterol

12) A patient comes into your clinic to seek your advice on ways to substitute meat in their diet with other sources of proteins. Which of the following are all alternative sources of proteins?

- ◯ Tofu, avocado, salmon
- ◯ Corn, brown rice, shrimp
- ◯ Bananas, peanuts, lotus root
- ◯ Carrots, brussel sprouts, mushrooms

13) What percentage of Americans do not meet the recommended daily vegetable intake?

- ◯ 10%
- ◯ 50%
- ◯ 70%
- ◯ 90%

14) Eggs labeled 100% Free Range indicate that the animals:

- ◯ Spent 100% of their time outdoors in an open field
- ◯ Had access to the outdoors
- ◯ Did not spend any time within chicken coops that were at 100% capacity
- ◯ Were not fed any artificial chicken fertilizer

15) A serving of protein is approximately equivalent to:

- ◯ Size of a palm
- ◯ Size of a fist
- ◯ Size of a thumb
- ◯ Size of a cupped hand

16) A common cited barrier to eating healthy is a lack of time to shop and prepare meals.

- ◯ True
- ◯ False

17) Fish oil is a good source of Omega-6 fatty acids and alpha-linoleic fatty acid .

- ◯ True
- ◯ False

18) When reading nutrition labels, what do total sugar values include?

- ◯ Only the sugars naturally found in the food
- ◯ Only the added sugars
- ◯ Only the artificially made sugars
- ◯ Sugars naturally found in the food and added sugars

19) What does the % Daily Value on a nutrition label indicate?

- ◯ The ratio of each different nutrient in the food, totaling to 100%
- ◯ How much a serving of the food contributes to your daily diet for each nutrient
- ◯ How much of this nutrient you need to consume a day to meet recommendations
- ◯ A way to calculate how many nutrients are in each serving size

20) Plant based milk (i.e., almond, rice, coconut, oat, hemp) contains the same nutrition content as dairy based milk.

- ◯ True
- ◯ False

For questions 21-23, match the following fatty acids to its best description. An answer choice can only be used once.

A. Omega 6 control blood sugar and lower blood pressure; Omega 3 can decrease triglycerides, increase HDL, and reduce inflammation; nuts are a good source

B. Lowers total and LDL cholesterol; avocado is a good source

C. Increases LDL, reduces HDL; still found in some fast foods

21) Monounsaturated Fatty Acids

- ◯ A
- ◯ B
- ◯ C

22) Polyunsaturated Fatty Acids

- ◯ A
- ◯ B
- ◯ C

23) Trans Fats

- ◯ A
- ◯ B
- ◯ C

Please adjust the slider as necessary for the next two questions.

24) How prepared do you currently feel about talking to patients in clinical settings about nutrition?

Not Prepared — Very Prepared

(Place a mark on the scale above)

25) How comfortable do you currently feel about advising patients in clinical settings on nutrition?

Not Comfortable — Very Comfortable

(Place a mark on the scale above)

The following set of questions were established by the Mayo Clinic* to assess for fatigue, burnout, and quality of life in medical students. Please answer them to the best of your ability. All answers will strictly remain anonymous and confidential.

26) During the past month, have you felt burned out from medical school?

◯ Yes ◯ No

27) During the past month, have you worried that medical school is hardening you emotionally?

◯ Yes ◯ No

28) During the past month, have you often been bothered by feeling down, depressed, or hopeless?

◯ Yes ◯ No

29) During the past month, have you fallen asleep while stopped in traffic or driving?

◯ Yes ◯ No

30) During the past month, have you felt that all things you had to do were piling up so high that you could not overcome them?

◯ Yes ◯ No

31) During the past month, have you been bothered by emotional problems (such as feeling anxious, depressed, or irritable)

○ Yes ○ No

32) During the past month, has your physical health interfered with your ability to do your daily work at home and/or away from home?

○ Yes ○ No

*Well-Being Index adapted from Mayo Clinic
Dyrbye, L.N., Szydlo, D.W., Downing, S.M. *et al.* Development and preliminary psychometric properties of a well-being index for medical students. *BMC Med Educ* **10**, 8 (2010). https://doi.org/10.1186/1472-6920-10-8
