# Supplementary material for: Nutrition Module: Addressing the Nutrition Education Gap in Undergraduate Medical Curricula via a Novel Approach
Source: Med Sci Educ. 2024 Jul 15;34(6):1361–7. doi: 10.1007/s40670-024-02114-9 (PMC11699193; doi:10.1007/s40670-024-02114-9)
Supplement: Supplementary file 3 — Post-Assessment (PDF 86 KB) [file 40670_2024_2114_MOESM3_ESM.pdf]

Post-Module Assessment

Thank you for your participation in this research study!

Your study ID is based on the model of your car and the last 4 digits of your cell phone number in all lowercase (e.g., camry8378).

1) Study ID

____

Select the best answer choice for assessment questions 2-21 without using resources.

2) What does the glycemic index of a carbohydrate tell you?

- ◯ How sweet the carbohydrate is
- ◯ The ratio of fiber to sugar in the carbohydrate
- ◯ How quickly the carbohydrate will cause an increase in blood glucose levels
- ◯ The ratio of fructose to glucose

3) For which of the following set of patients is it especially important for them to understand glycemic index?

- ◯ Patients with sleep disorders
- ◯ Patients older than 60 years
- ◯ Patients with diabetes
- ◯ Patients with high blood pressure

4) For a product to be marked 100% whole grain, what is the minimum requirement for grams of grain per serving?

- ◯ 11g
- ◯ 16g
- ◯ 21g
- ◯ 26g

5) Which of the following is NOT an example of polyunsaturated fats?

- ◯ Salmon
- ◯ Avocado
- ◯ Almonds
- ◯ Flaxseed

6) White bread is an example of a carbohydrate with high glycemic index.

- ◯ True
- ◯ False

7) Frozen fruits contain less nutritional value than fresh fruits.

- ◯ True
- ◯ False

8) It is recommended to choose foods that are:

- ◯ Higher in %Daily Value for Dietary Fiber and Vitamin D
- ◯ Higher in %Daily Value for Calcium, Iron, and Potassium
- ◯ Lower in %Daily Value for Saturated Fat, Sodium, and Added Sugars
- ◯ All of above

9) The benefits of consuming fiber include:

- ◯ Preventing diarrhea
- ◯ Weight gain
- ◯ Lowering the risk of heart disease
- ◯ Increasing your HDL cholesterol

10) A patient comes into your clinic to seek your advice on ways to substitute meat in their diet with other sources of proteins. Which of the following are all alternative sources of proteins?

- ◯ Tofu, avocado, salmon
- ◯ Corn, brown rice, shrimp
- ◯ Bananas, peanuts, lotus root
- ◯ Carrots, brussel sprouts, mushrooms

11) What percentage of Americans do not meet the recommended daily vegetable intake?

- ◯ 10%
- ◯ 50%
- ◯ 70%
- ◯ 90%

12) Eggs labeled 100% Free Range indicate that the animals:

- ◯ Spent 100% of their time outdoors in an open field
- ◯ Had access to the outdoors
- ◯ Did not spend any time within chicken coops that were at 100% capacity
- ◯ Were not fed any artificial chicken fertilizer

13) A serving of protein is approximately equivalent to:

- ◯ Size of a palm
- ◯ Size of a fist
- ◯ Size of a thumb
- ◯ Size of a cupped hand

14) A common cited barrier to eating healthy is a lack of time to shop and prepare meals.

- ◯ True
- ◯ False

15) Fish oil is a good source of Omega-6 fatty acids and alpha-linoleic fatty acid .

- ◯ True
- ◯ False

16) When reading nutrition labels, what do total sugar values include?

- ◯ Only the sugars naturally found in the food
- ◯ Only the added sugars
- ◯ Only the artificially made sugars
- ◯ Sugars naturally found in the food and added sugars

17) What does the % Daily Value on a nutrition label indicate?

- ◯ The ratio of each different nutrient in the food, totaling to 100%
- ◯ How much a serving of the food contributes to your daily diet for each nutrient
- ◯ How much of this nutrient you need to consume a day to meet recommendations
- ◯ A way to calculate how many nutrients are in each serving size

18) Plant based milk (i.e., almond, rice, coconut, oat, hemp) contains the same nutrition content as dairy based milk.

- ◯ True
- ◯ False

For questions 21-23, match the following fatty acids to its best description. An answer choice can only be used once.

A. Omega 6 control blood sugar and lower blood pressure; Omega 3 can decrease triglycerides, increase HDL, and reduce inflammation; nuts are a good source

B. Lowers total and LDL cholesterol; avocado is a good source

C. Increases LDL, reduces HDL; still found in some fast foods

19) Monounsaturated Fatty Acids

- ◯ A
- ◯ B
- ◯ C

20) Polyunsaturated Fatty Acids

- ◯ A
- ◯ B
- ◯ C

21) Trans Fats

- ◯ A
- ◯ B
- ◯ C
