# Supplementary material for: Nutrition Module: Addressing the Nutrition Education Gap in Undergraduate Medical Curricula via a Novel Approach
Source: Med Sci Educ. 2024 Jul 15;34(6):1361–7. doi: 10.1007/s40670-024-02114-9 (PMC11699193; doi:10.1007/s40670-024-02114-9)
Supplement: Supplementary file 4 — Follow Up Assessment (PDF 180 KB) [file 40670_2024_2114_MOESM4_ESM.pdf]

Follow Up Assessment

Thank you for your participation in this research study! Please complete the follow up assessment, and successful completion will mark the end of your participation in this research project.

Your study ID is based on the model of your car and the last 4 digits of your cell phone number in all lowercase (e.g., camry8378).

1) Study ID

____

Select the best answer choice for assessment questions 2-21 without using resources.

2) What does the glycemic index of a carbohydrate tell you?
- ◯ How sweet the carbohydrate is
- ◯ The ratio of fiber to sugar in the carbohydrate
- ◯ How quickly the carbohydrate will cause an increase in blood glucose levels
- ◯ The ratio of fructose to glucose

3) For which of the following set of patients is it especially important for them to understand glycemic index?
- ◯ Patients with sleep disorders
- ◯ Patients older than 60 years
- ◯ Patients with diabetes
- ◯ Patients with high blood pressure

4) For a product to be marked 100% whole grain, what is the minimum requirement for grams of grain per serving?
- ◯ 11g
- ◯ 16g
- ◯ 21g
- ◯ 26g

5) Which of the following is NOT an example of polyunsaturated fats?
- ◯ Salmon
- ◯ Avocado
- ◯ Almonds
- ◯ Flaxseed

6) White bread is an example of a carbohydrate with high glycemic index.
- ◯ True
- ◯ False

7) Frozen fruits contain less nutritional value than fresh fruits.
- ◯ True
- ◯ False

8) It is recommended to choose foods that are:
- ◯ Higher in %Daily Value for Dietary Fiber and Vitamin D
- ◯ Higher in %Daily Value for Calcium, Iron, and Potassium
- ◯ Lower in %Daily Value for Saturated Fat, Sodium, and Added Sugars
- ◯ All of above

9) The benefits of consuming fiber include:
- ◯ Preventing diarrhea
- ◯ Weight gain
- ◯ Lowering the risk of heart disease
- ◯ Increasing your HDL cholesterol

10) A patient comes into your clinic to seek your advice on ways to substitute meat in their diet with other sources of proteins. Which of the following are all alternative sources of proteins?

- ◯ Tofu, avocado, salmon
- ◯ Corn, brown rice, shrimp
- ◯ Bananas, peanuts, lotus root
- ◯ Carrots, brussel sprouts, mushrooms

11) What percentage of Americans do not meet the recommended daily vegetable intake?

- ◯ 10%
- ◯ 50%
- ◯ 70%
- ◯ 90%

12) Eggs labeled 100% Free Range indicate that the animals:

- ◯ Spent 100% of their time outdoors in an open field
- ◯ Had access to the outdoors
- ◯ Did not spend any time within chicken coops that were at 100% capacity
- ◯ Were not fed any artificial chicken fertilizer

13) A serving of protein is approximately equivalent to:

- ◯ Size of a palm
- ◯ Size of a fist
- ◯ Size of a thumb
- ◯ Size of a cupped hand

14) A common cited barrier to eating healthy is a lack of time to shop and prepare meals.

- ◯ True
- ◯ False

15) Fish oil is a good source of Omega-6 fatty acids and alpha-linoleic fatty acid .

- ◯ True
- ◯ False

16) When reading nutrition labels, what do total sugar values include?

- ◯ Only the sugars naturally found in the food
- ◯ Only the added sugars
- ◯ Only the artificially made sugars
- ◯ Sugars naturally found in the food and added sugars

17) What does the % Daily Value on a nutrition label indicate?

- ◯ The ratio of each different nutrient in the food, totaling to 100%
- ◯ How much a serving of the food contributes to your daily diet for each nutrient
- ◯ How much of this nutrient you need to consume a day to meet recommendations
- ◯ A way to calculate how many nutrients are in each serving size

18) Plant based milk (i.e., almond, rice, coconut, oat, hemp) contains the same nutrition content as dairy based milk.

- ◯ True
- ◯ False

For questions 21-23, match the following fatty acids to its best description. An answer choice can only be used once.

A. Omega 6 control blood sugar and lower blood pressure; Omega 3 can decrease triglycerides, increase HDL, and reduce inflammation; nuts are a good source

B. Lowers total and LDL cholesterol; avocado is a good source

C. Increases LDL, reduces HDL; still found in some fast foods

19) Monounsaturated Fatty Acids

- ◯ A
- ◯ B
- ◯ C

20) Polyunsaturated Fatty Acids

◯ A
◯ B
◯ C

21) Trans Fats

◯ A
◯ B
◯ C

Please adjust the slider as necessary for the next two questions.

22) How prepared do you currently feel about talking to patients in clinical settings about nutrition?

Not Prepared — Very Prepared

(Place a mark on the scale above)

23) How comfortable do you currently feel about advising patients in clinical settings on nutrition?

Not Comfortable — Very Comfortable

(Place a mark on the scale above)

The following set of questions were established by the Mayo Clinic* to assess for fatigue, burnout, and quality of life in medical students. Please answer them to the best of your ability. All answers will strictly remain anonymous and confidential.

24) During the past month, have you felt burned out from medical school?

◯ Yes ◯ No

25) During the past month, have you worried that medical school is hardening you emotionally?

◯ Yes ◯ No

26) During the past month, have you often been bothered by feeling down, depressed, or hopeless?

◯ Yes ◯ No

27) During the past month, have you fallen asleep while stopped in traffic or driving?

◯ Yes ◯ No

28) During the past month, have you felt that all things you had to do were piling up so high that you could not overcome them?

◯ Yes ◯ No

29) During the past month, have you been bothered by emotional problems (such as feeling anxious, depressed, or irritable)

◯ Yes ◯ No

30) During the past month, has your physical health interfered with your ability to do your daily work at home and/or away from home?

◯ Yes ◯ No

*Well-Being Index adapted from Mayo Clinic
Dyrbye, L.N., Szydlo, D.W., Downing, S.M. *et al.* Development and preliminary psychometric properties of a well-being index for medical students. *BMC Med Educ* **10**, 8 (2010). https://doi.org/10.1186/1472-6920-10-8

Please rate how strongly you agree or disagree with each statement below.

31) As a result of the nutrition modules, I have changed my dietary habits.

- ◯ strongly disagree
- ◯ disagree
- ◯ neutral
- ◯ agree
- ◯ strongly agree

32) The nutrition modules did not influence my dietary habits.

- ◯ strongly disagree
- ◯ disagree
- ◯ neutral
- ◯ agree
- ◯ strongly agree

33) The nutrition modules positively influenced my dietary habits.

- ◯ strongly disagree
- ◯ disagree
- ◯ neutral
- ◯ agree
- ◯ strongly agree

34) My personal dietary habits meet the recommended nutritional intake.

- ◯ strongly disagree
- ◯ disagree
- ◯ neutral
- ◯ agree
- ◯ strongly agree

35) I feel confident that I have healthy dietary habits.

- ◯ strongly disagree
- ◯ disagree
- ◯ neutral
- ◯ agree
- ◯ strongly agree
