# Supplementary material for: Nutrition Module: Addressing the Nutrition Education Gap in Undergraduate Medical Curricula via a Novel Approach
Source: Med Sci Educ. 2024 Jul 15;34(6):1361–7. doi: 10.1007/s40670-024-02114-9 (PMC11699193; doi:10.1007/s40670-024-02114-9)
Supplement: Supplementary file 5 — Nutrition Module (DOCX 15 KB) [file 40670_2024_2114_MOESM5_ESM.docx]

Nutrition Module Link

<https://360.articulate.com/review/content/f13d9854-e166-4505-81aa-51a1198ecece/review>
